# Incidence and risk factors of hearing loss in patients with Turner Syndrome

**DOI:** 10.3389/fpubh.2023.1076812

**Published:** 2023-03-14

**Authors:** Huijia Lin, Xiaoya Wang, Shuang Qin, Fanglan Luo, Yingmei Cen, Gendie E. Lash, Li Li

**Affiliations:** ^1^Department of Obstetrics and Gynecology, Guangzhou Women and Children's Medical Center, Guangzhou Medical University, Guangzhou, China; ^2^Department of Ear, Nose, and Throat, Guangzhou Women and Children's Medical Center, Guangzhou Medical University, Guangzhou, China; ^3^Guangzhou Institute of Pediatrics, Guangzhou Women and Children's Medical Center, Guangzhou Medical University, Guangdong Provincial Clinical Research Center for Child Health, Guangzhou, China

**Keywords:** Turner Syndrome, hearing loss, age, karyotype, audiological examination

## Abstract

**Background:**

Hearing loss (HL) is one of the main medical complications for Turner Syndrome (TS) patients, with an earlier presentation and higher incidence than normal women. However, the etiology of HL in TS is unclear. The aim of this study was to investigate the hearing status of TS patients in China and the influencing factors, so as to provide a theoretical basis for early intervention treatment for TS patients with HL.

**Methods:**

In total 46 female patients aged 14–32 diagnosed with TS received tympanic membrane and audiological examinations, including pure tone audiometry and tympanometry. In addition, the effects of karyotype, sex hormone levels, thyroid function, insulin, blood lipids, bone mineral density, age and other factors on hearing levels were analyzed, and the possible risk factors associated with HL in TS patients were explored.

**Results:**

In 9 patients (19.6%) had HL, including 1 (2.2%) with mild conductive hearing loss, 5 (10.9%) with mild sensorineural hearing loss, 3 (6.5%) with moderate sensorineural hearing loss. TS is often associated with age-related mid-frequency and high-frequency HL, and the incidence of HL increases with age. Compared with other karyotypes, patients with 45, X haplotype have an increased risk of mid-frequency HL.

**Conclusions:**

Therefore, karyotype may be a predictor of hearing problems in TS.

## 1. Introduction

Turner Syndrome (TS) is one of the most common human chromosomal abnormalities and is a major cause of short stature and ovarian insufficiency in women, which is caused by partial or complete loss of the X chromosome. The most common karyotype of TS is 45, X, accounting for about 30 to 40% of all karyotypes, followed by mosaic patterns (45, X/46, XX), accounting for about 20–30%, and the rest are X chromosome abnormalities (1, 2). The incidence of TS in live born girls is about 1/2,000–1/2,500, and the incidence may vary in different countries and regions (3). In recent years, with the wide application of prenatal diagnosis technology, the age of diagnosis of TS has been advanced, but most TS patients are usually diagnosed in childhood or adolescence due to short stature or primary gonadal dysplasia, and the average age of diagnosis is 15 years old (3).

In addition to gonadal dysgenesis and short stature, with the increase of age, TS is often accompanied by a variety of complications, such as cardiovascular disease, autoimmune disease, metabolic disease, osteoporosis, and neurocognitive deficits (4). Hearing loss (HL) is one of the main medical problems for girls and women with TS and has a negative impact on the health and quality of life for these patients. A previous study demonstrated that only 48% of Swedish women aged 25 to 38 with TS had normal hearing, while 45% had mild HL and 7% had moderate or severe HL (5). On average, the hearing threshold of TS women is equivalent to the hearing threshold of women in the general population 20 years older than their actual age (5). The specific etiology of HL caused by TS has not been extensively studied, it may be the influence of estrogen, chromosome and gene abnormalities, or their combination (6, 7). Due to ovarian hypoplasia, TS patients have little or no endogenous estrogen production, and estrogen has a certain protective effect on hearing (8, 9). In addition, some genes on the short arm of the X chromosome may have a regulatory effect on hearing function, and their deletion may cause abnormal craniofacial development and auricle abnormalities, which may be related to the prolonged cell cycle caused by chromosomal abnormalities (7, 10). Some patients with TS may have abnormalities of the Eustachian tube and the skull base, repeated occurrence of otitis media, middle ear effusion, and cholesteatoma can lead to middle ear destruction and progressive HL (7, 11).

TS patients often also have endocrine abnormalities, metabolic disease and abnormal bone metabolism. Studies have shown that low bone mineral density (BMD), thyroid dysfunction and metabolic syndrome are risk factors for HL (12–17), and timely supplementation of thyroid hormone or lowering of blood lipids can improve hearing. However, it is not known if this association also exists in women with TS.

At present, the treatment of TS is mainly growth-promoting therapy, inducing and maintaining the development of secondary sexual characteristics, preventing and curing osteoporosis. Less attention has been paid to the ear health of TS patients, which delays optimal treatment time. Early diagnosis and proper management of associated hearing abnormalities are important to minimize the adverse effects on learning and social functions and improve quality of life. Here, we analyzed the incidence, types and risk factors of HL in TS patients in China, with an aim to improve the prevention and treatment of TS ear diseases and improve the quality of life of women living with TS.

## 2. Materials and methods

### 2.1. Clinical characteristics

Forty-six female patients diagnosed with TS in the Department of Gynecology and Endocrinology, Guangzhou Women and Children's Medical Center affiliated to Guangzhou Medical University were recruited and confirmed by chromosome karyotype analysis of their peripheral blood as previously described (17). Patients who also had another genetic disease (or another related syndrome) affecting hearing and/or refused to cooperate and were unable to perform audiometry testing were excluded. All patients denied a history of ear trauma, noise exposure, ototoxic drug use, smoking and drinking history. General information and clinical data of the patients were collected, including height, weight, chromosome karyotype, blood lipid concentrations, thyroid function, BMD, and hormone treatment status. This study was approved by the Ethics Committee of Guangzhou Women and Children's Medical Center (2016042019), and all participants or their guardians signed an informed consent form.

### 2.2. Diagnostic criteria

#### 2.2.1. Turner Syndrome diagnostic criteria

The diagnostic criteria for TS included a karyotype containing one X chromosome and complete or partial absence of the second sex chromosome, associated with one or more typical clinical manifestations of TS, including short stature, hypoplastic secondary sexual characteristics, primary or secondary amenorrhea during adolescence, webbed neck, elbow valgus, cardiovascular and urinary system and other malformations (3).

#### 2.2.2. Diagnostic criteria for audiology

According to the 2021 World Health Organization's hearing loss classification standards, using the pure tone average air conduct threshold between 500, 1,000, 2,000, and 4,000 Hz frequencies as the hearing threshold, HL is defined as having hearing thresholds greater (worse) than 20 decibel (dB) alteration at 6 and/or 8 kHz, and the PTA value obtained by average air conduction thresholds between 500, 1,000, 2,000, and 4,000 Hz frequencies is used as the basis for classifying hearing loss levels: (1) Normal hearing: less than 20 dB; (2) Mild HL: 20 to <35 dB; (3) Moderate HL: 35 to <50 dB; (4) Moderately severe HL: 50 to <65 dB; (5) Severe HL: 65 to <80 dB; (6) Profound HL: 80 to <95 dB; (7) Complete or total HL: 95 dB or greater; and (8) Unilateral: <20 dB in the better ear, 35 dB or greater in the worse ear. Using the PTA air-bone gap (PTA-ABG) and bone conduction pure tone average (PTA), HL is divided into conductive, sensorineural and mixed hearing loss.

According to the pressure variations exerted in the outer ear canal 19, the tympanic impedance is divided into Type A (normal, with a symmetric wave), Type B (rounded or flat curve), Type C (low wave with leftward shift) and Type Ad (very high wave) (18, 19).

#### 2.2.3. Diagnostic criteria for patient investigations

All TS patients received diagnosis and treatment in Guangzhou Women and Children's Medical Center, and underwent detailed consultation (including past and present medical history), tympanic membrane and audiological examination by otorhinolaryngology specialists. Pure tone audiometry was performed with GSI AudioStarPro in the audiometry room with background noise less than 25 dB. Each frequency was repeated three times, and the air conducting threshold was recorded at 125, 250, 1,000, 2,000, 4,000, and 8,000 Hz frequencies, respectively. Bone conduction thresholds were recorded at 125, 250, 1,000, 2,000, and 4,000 Hz respectively. Tympanometry was performed with a GSI TympStar middle ear analyzer, with probe tone set to a frequency of 226 Hz. Peripheral blood was collected into inert separating gelatinizing tubes, serum collected and stored at −20°C until required for analysis. Thyroid stimulating hormone (TSH), free thyroxine (FT4), thyroid peroxidase antibody (TPO-Ab), triglyceride (TG), total cholesterol (TC), low- density lipoprotein (LDL), and high-density lipoprotein (HDL) were measured in the serum of all subjects. Thyroid function was measured by the electron chemiluminescence immunoassay in the Abbott I2000 analyzer, and the blood lipid levels of all subjects were measured by the Hitachi 7600-200 analyzer. Body fat, waist:hip ratio and BMD in different sites were measured by dual energy X-ray absorptiometry (DXA) using a Lunar DXA densitometer (Lunar Corporation, Madison WI, U.S.A.).

### 2.3. Statistical analysis

The SPSS 26.0 software package was used for statistical analysis of all data. The measurement data with normal distribution is represented by mean ± standard deviation, and the comparison between the two groups was performed by test. Counting data are expressed as the number of cases and percentages and compared using chi-square test or Fisher's exact test. Linear regression analysis was used to explore causal relationships. *P* < 0.05 was considered as statistically significant.

## 3. Results

### 3.1. Patient characteristics

A total of 46 patients aged 14–32 years with a diagnosis of TS were included in the study, and the basic characteristics of the study participants is shown in Table 1. In total of 18 patients had a chromosome karyotype of 45, X, and the rest had other chromosome karyotypes, including mosaicism, X chromosome aberration and contained Y chromosome, among which 3 patients had no deletion of the SHOX gene. In line with existing guidelines for the treatment of TS, all the participants received hormone replacement treatment (HRT).

**Table 1:** Clinical characteristics of the study participants.

**Subject**	**Value**
Age (years)	20.57 ± 0.67
Height (m)	1.50 ± 0.01
Weight (kg)	51.94 ± 1.43
BMI (kg/m^2^)	23.13 ± 0.58
Age at diagnosis (years)	14.35 ± 5.36
Duration of GHT	4.47 ± 3.18
Duration of HRT	3.70 ± 3.28
**Karyotype**	
Monosomy X (45, X) [*n* (%)]	18 (39.1%)
Other alterations [*n* (%)]	28 (60.9%)
**Short stature homeobox**	
Exists [*n* (%)]	3 (6.5%)
Missing [*n* (%)]	43 (93.5%)
FSH (mIU/ml)	34.21 (8.89, 67.16)
LH (mIU/ml)	7.99 (3.09, 17.52)
E2 (pmol/l)	93.00 (37.00, 134.50)
INS (Uu/mL)	7.50 (5.78, 10.15)
TC (mmol/l)	5.27 ± 0.16
TG (mmol/l)	1.62 ± 0.11
HDL-C (mmol/l)	1.61 ± 0.05
LDL-C (mmol/l)	2.98 ± 0.13
TG/HDL-C	1.06 ± 0.83
TSH (mIU/l)	2.16 (1.75, 2.95)
FT4 (pmol/l)	13.46 ± 0.24
TPO-Ab (IU/ml)	1.46 (0.08, 247.68)
WHR	0.93 ± 0.02
BF (%)	35.16 ± 0.90
Whole Body BMD (g/cm2)	0.89 ± 0.01
Lumbar Spine BMD (g/cm2)	0.77 ± 0.02
Femur Neck BMD (g/cm2)	0.65 ± 0.02
Total Hip BMD (g/cm2)	0.65 ± 0.02

GHT, Growth hormone treatment; HRT, Hormone replacement treatment; WHR, Waist hip ratio; BF, Body fat; BMD, Bone mineral density.

### 3.2. Audiological examination

A total of 92 ears of 46 patients underwent pure tone audiometry and tympanometry (Table 2). It was found that 9 (19.6%) had HL, including 1 (2.2%) with mild conductive hearing loss, 5 (10.9%) with mild sensorineural hearing loss, 3 (6.5%) with moderate sensorineural hearing loss, but none had moderately severe or higher hearing loss. In addition, 6 (13.0%) only had high-frequency hearing loss in the PTA; 36 (78.2%) had “A” type tympanogram, 7 (15.2%) “Ad” type, 1 (2.2%) “B” type, and 2 (4.4%) “C” type. Compared with other karyotypes (including mosaicism, chromosomal abnormality, and Y chromosome-containing type), 45, X haplotype patients had higher hearing thresholds at 1, 2, and 4 KHz, and hearing thresholds at 1 and 2 KHz were higher than the normal level (*P* < 0.05) (Table 3). However, there was no significant correlation between chromosome karyotype and the type and degree of HL (Table 4). In addition, three TS patients without *SHOX* gene deletion had normal audiological examinations, but *SHOX* gene abnormalities had no association with the pure tone audiometry and tympanogram results (data not shown).

**Table 2:** Audiological tests for TS of the study.

**Subject**	**Value**
**Types of hearing loss**	
Normal	31 (67.4%)
Conductive	1 (2.2%)
Sensorineural	8 (17.4%)
Alteration at 6 and/or 8 kHz	6 (13.0%)
**Degree of loss**	
Normal	37 (80.5%)
Mild	6 (13.0%)
Moderate	3 (6.5%)
**Tympanogram**	
A	36 (78.2%)
Ad	7 (15.2%)
B	1 (2.2%)
C	2 (4.4%)

**Table 3:** Threshold of hearing at different frequencies for different karyotypes.

**Frequencies**	**Monosomy X (*n =* 18)**	**Other alterations (*n =* 28)**	***P* value**
125 Hz	19.52 ± 10.23	22.06 ± 12.13	0.43
250 Hz	18.61 ± 11.44	17.95 ± 10.78	0.78
500 Hz	16.81 ± 11.72	15.45 ± 12.33	0.60
1,000 Hz	21.25 ± 13.60	14.46 ± 14.23	0.03^*^
2,000 Hz	21.67 ± 15.72	13.30 ± 13.43	0.01^*^
4,000 Hz	19.31 ± 17.53	12.14 ± 11.59	0.04^*^
8,000 Hz	22.50 ± 20.99	15.45 ± 11.81	0.11

Other alterations: mosaicism, X chromosome aberration and contained Y chromosome. ^*^Denotes significant differences.

**Table 4:** Distribution of chromosomal karyotypes and genes with types and degrees of hearing loss in patients with Turner Syndrome.

**Type of hearing loss**	**Karyotype**	
	**Monosomy X (*****n** =* **18)**	**Other alterations (*****n** =* **28)**
Conductive	0 (0.0%)	1 (100.0%)
Sensorineural	5 (62.5%)	3 (37.5%)
Alteration at 6 and/or 8 kHz	2 (33.3%)	4 (66.7%)
**Degree of hearing loss**		
Mild	3 (50.0%)	3 (50.0%)
Moderate	2 (66.7%)	1 (33.3%)
**Tympanogram**		
A	15 (41.7%)	21 (58.3%)
Ad	2 (28.6%)	6 (71.4%)
B	0 (0.0%)	1 (100.0%)
C	1 (50.0%)	1 (50.0%)

Comparisons were done by Fisher's Exact Test, and *P* > 0.05.

### 3.3. Associated factors for hearing loss in TS subjects

**Figure 1:**
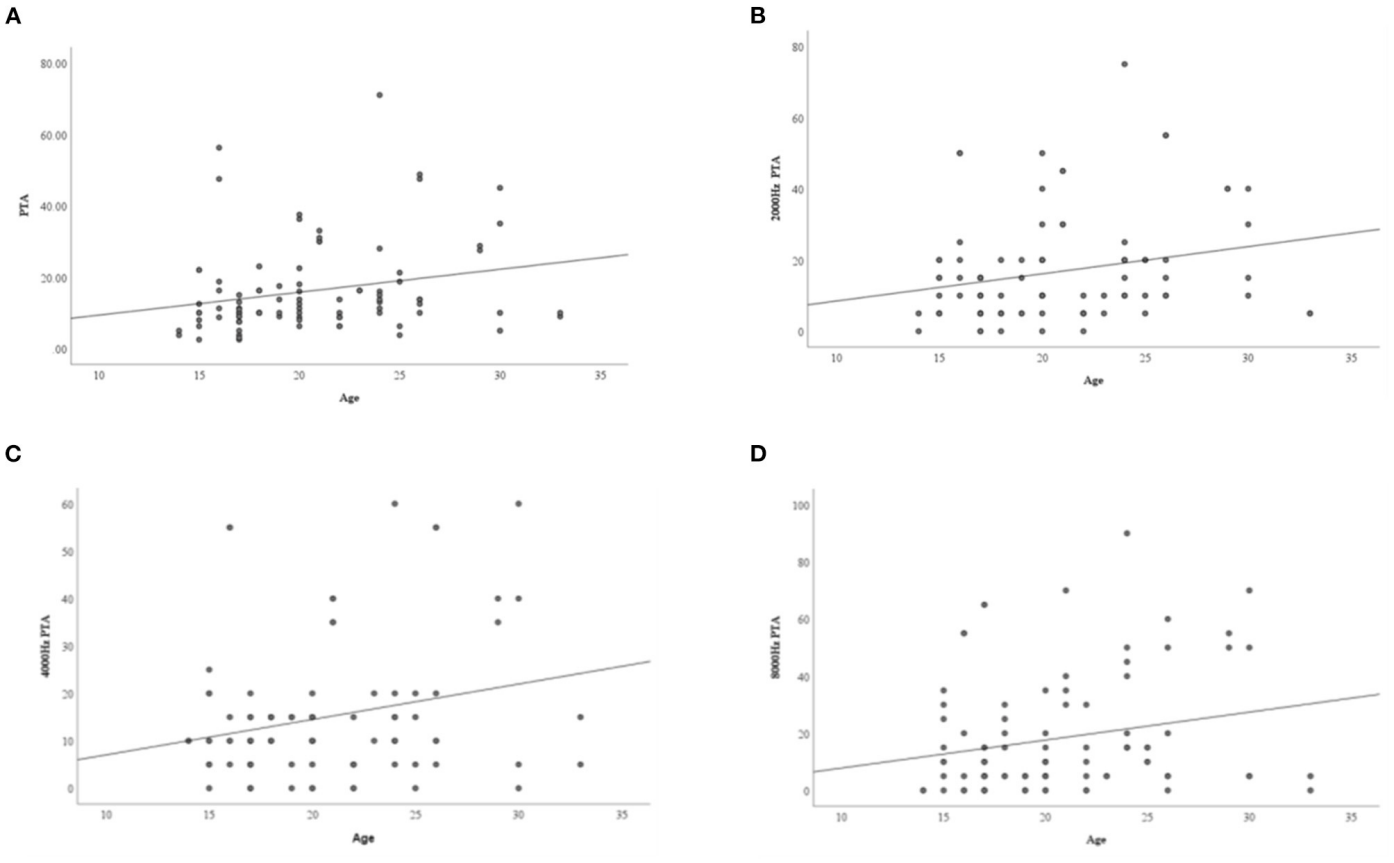
The relationship between age and PTA at different frequencies. **(A)** Average hearing threshold increases with age; **(B)** 2,000 Hz hearing threshold increases with age; **(C)** 4,000 Hz hearing threshold increases with age; **(D)** 8,000 Hz hearing threshold increases with age.

The results showed that there were statistically significant differences in age and height between the normal hearing group and hearing loss group (as defined by PTA) (*P* < 0.05) (Table 5). The age of the hearing loss group was higher than that of the normal hearing group, while the height of the hearing loss group was lower than that of the normal hearing group. Obesity, insulin resistance, blood lipid levels, thyroid function, estrogen levels, and BMD were not associated with HL in TS, and growth hormone treatment (GHT) and hormone replacement treatment (HRT) were not significantly associated with hearing status in TS. Linear regression analysis was performed on age and pure tone average of each frequency, and it was found that with an increase in age, the hearing thresholds and average hearing thresholds of 2, 4, and 8 KHz increased (*P* < 0.05) (Table 6, Figure 1).

**Table 5:** Factors associated with hearing loss.

**Subject**	**Normal audiometry**	**Altered audiometry**	***P* value**
Age (years)	19.89 ± 4.40	23.44 ± 4.48	0.04^*^
Height (m)	1.51 ± 0.06	1.46 ± 0.06	0.03^*^
Weight (kg)	52.74 ± 9.31	48.65 ± 11.00	0.26
BMI (kg/m2)	23.24 ± 3.90	22.65 ± 4.18	0.69
BF (%)	34.46 ± 4.93	37.45 ± 5.90	0.16
WHR	0.94 ±0.13	0.91 ± 0.12	0.61
Whole Body BMD (g/cm^2^)	0.89 ± 0.07	0.87 ± 0.11	0.06
Lumbar Spine BMD (g/cm^2^)	0.77 ± 0.09	0.76 ± 0.14	0.95
Femur Neck BMD (g/cm^2^)	0.64 ± 0.11	0.66 ± 0.16	0.65
Total Hip BMD (g/cm^2^)	0.72 ± 0.11	0.73 ± 0.12	0.80
FSH (mIU/ml)	35.50 (9.41, 72.78)	13.82 (6.86, 45.50)	0.23
LH (mIU/ml)	8.60 (3.04, 18.66)	6.63 (2.72, 12.73)	0.26
E2 (pmol/l)	92.0 (37.00, 137.00)	100.20 (37.00, 152.50)	0.84
TSH (mIU/l)	2.20 (1.66, 3.19)	1.94 (1.80, 2.85)	0.68
FT4 (pmol/l)	13.32 ± 1.53	14.07 ± 1.94	0.21
TPO-Ab (IU/ml)	8.99 (0.08, 274.92)	0.44 (0.06, 59.27)	0.48
TC (mmol/l)	5.11 ± 0.73	5.90 ± 1.82	0.23
TG (mmol/l)	1.59 ± 0.69	1.71 ± 1.02	0.67
HDL-C (mmol/l)	1.59 ± 0.26	1.70 ± 0.52	0.55
LDL-C (mmol/l)	2.93 ± 0.58	3.20 ± 1.60	0.63
TG/HDL-C	1.03 ± 0.48	1.18 ± 0.85	0.64
INS (Uu/mL)	7.90 (5.67, 11.30)	7.50 (6.50, 8.40)	0.77
Duration of GHT	2.31 ± 3.10	1.44 ± 3.36	0.46
Duration of HRT	3.24 ± 2.94	5.56 ± 4.10	0.06

GHT, Growth hormone treatment; HRT, Hormone replacement treatment; WHR, Waist hip ratio; BF, Body fat; BMD, Bone mineral density. ^*^Denotes significant differences.

**Table 6:** Relationship between age and PTA at different frequencies.

	**PTA/age**	**2,000 Hz PTA/age**	**4,000 Hz PTA/age**	**8,000 Hz PTA/age**
*R*-Value	0.23	0.23	0.23	0.22
*P*-Value	0.02^*^	0.02^*^	0.02^*^	0.04^*^

^*^Denotes significant differences.

## 4. Discussion

Due to complete or partial deletion of the X chromosome, patients with TS often have associated developmental abnormalities and malformations, and different cytogenetic changes can lead to clinical heterogeneity. In addition to gonadal dysgenesis and short stature, with the increase of age, TS women often encounter a variety of complications, such as cardiovascular disease, autoimmune disease, metabolic disease, osteoporosis, and neurocognitive deficits (4).

Hearing problems can occur at all ages in TS patients, and the incidence is higher than that of normal people (5, 20–22). The types of HL in TS patients included conductive hearing loss (CHL), sensorineural hearing loss (SNHL), and mixed hearing loss (MHL). The study found that the incidence of TS combined with HL varies greatly in different regions and populations. The occurrence of CHL is related to pharyngeal canal and craniofacial dysplasia (23). The incidence of CHL in TS patients is about 6–43%, and it is often secondary to chronic or recurrent otitis media (23–25), which is more common in childhood and adolescence, and continues to develop into adulthood (23). At present, the diagnostic criteria and definition of the intermediate frequency range of SNHL vary, and the incidence of SNHL varies greatly among different studies. Some scholars further distinguished mid-frequency hearing loss (MFHL) or high frequency hearing loss (HFHL) as distinct forms of HL. Chan et al. (26) stipulated that the intermediate frequency was between 1–2 k Hz, while Verver et al. (27) believed that the intermediate frequency was between 0.5–2 k Hz. Studies have found that the incidence of SNHL in women with TS is about 9–66%, often presenting as HFHL and/or MFHL. The pure tone audiometry shows a gradual descent and steep descent curve, which is similar to age-related HL, but the onset age is much younger and the progression rate is much faster (20). This progressive HL is more common in people with a history of recurrent ear infections in the early stage, and is usually associated with a defect in the outer helix hair cells in the middle and lower part of the cochlea, which is aggravated with age (28). MHL occurs in 3–13% of TS patients, and is more common in patients aged 30 years and above (10, 28). In this study, 46 TS patients aged 14–32 years were studied. The incidence of CHL was 2.2%, SNHL 17.4%, HFHL 13.0%, and MHL was not found, presenting with age-related HL. Compared with previous studies, the incidence of various types of HL in TS patients in this study was lower, which may be related to race, age composition ratio and small sample size. In addition, we did not compare HL levels with the normal population, however based on relevant reference ranges levels of HL were higher in our TS population.

Young and middle-aged women with TS often present with progressive HL, which intensifies rapidly in adulthood (20–22). HL is mainly composed of two causes: MFHL, which may be related to genetic factors; and age-related HFHL, which may be affected by estrogen deficiency (21). The incidence of HL in TS women was much higher than that in the general population. Regardless of initial age, hearing level, karyotype, or the presence or absence of MFHL, TS patients had a similar rate of de-cline as normal women aged 70–90 years, especially in the high-frequency region, with a decrease of 0.8–2.2 dB per year (21). High-frequency SNHL is common in TS patients (5, 29). Compared with chimeric TS patients, 45, X haplotype TS patients had a more significant age-dependent increase in high-frequency hearing threshold (29). Morimoto et al. (29) studied the hearing status of 33 female TS patients aged 8–40 years and found that more than 60% of TS patients suffered from high-frequency SNHL, and the incidence increased with age, and is usually associated with defect in the outer helix hair cells in the middle and lower part of the cochlea (30, 31). Animal experiments (32, 33) showed that “Turner mice” had ear and hearing problems similar to those of TS women, SNHL was usually accompanied by MFHL and early HFHL, and the loss of outer hair cells was obvious in the cochlear basal turn of Turner mice, and the expression of estrogen receptor in outer hair cells of the helical organ was weakened. These results suggest that estrogen and its receptor have protective effects on the development of cochlea. The results of our study also found that TS patients presented with age-related HL in middle and high frequency, and 45, X karyotypes were more common, indicating that HL was related to age and chromosome karyotype. Given the age related incidence of HL in TS patients we would recommend yearly follow up for these patients, in line with Chinese guidelines for people at high risk of HL.

Previous studies have observed that karyotype is related to the type and degree of HL in TS patients. It has been reported that those with a TS karyotype of 45, X are more likely to suffer from SNHL, compared to mosaicism or structural anomaly, patients with a monosomy 45, X or isochromosome (both have a total deletion of the short (p) arm of the X-chromosome) had more pronounced HL, which supports the hypothesis that hearing can be affected due to the loss of the X chromosome p arm, and the loss of different parts of the short arm of the X chromosome causes different phenotypes of HL (27, 33, 34). Barrenas et al. (35) found that the incidence of SNHL and auricular abnormalities increased significantly with the proportion of 45, X cells in TS individuals, suggesting that the disease was not only caused by specific X chromosome deletion, but also related to the cell cycle extension caused by chromosome aberration itself. Hearing is affected by dysfunctions of the outer, middle and inner ears caused by growth disorders during development. However, Bazilio et al. (28), Bois et al. (36) found that there was no statistically significant correlation between karyotype and the type or degree of HL. This study explored the relationship between chromosome karyotype and hearing in TS patients, and it was found that chromosome karyotype was unrelated to the type and degree of HL. Compared with other chromosome karyotypes (including chimeric type, abnormal chromosome structure type and body type with Y staining), hearing frequency of 45, X haplotypes increased at 1,000, 2,000, and 4,000 Hz. It suggests that the HL of TS may be related to the short arm deletion of X chromosome. In addition, our study found that audiology was normal in the 3 TS patients without deletion of *SHOX* gene, supporting the hypothesis that the absence of growth regulation genes, such as *SHOX* gene, may be associated with the occurrence of early HL, suggesting that karyotype or *SHOX* gene can be used as a predictor of hearing problems in TS.

Studies have shown that the incidence of HL with TS women is much higher than that in women of the same age, and age-related hearing impairment may be affected by estrogen deficiency (10, 21, 29). It has been observed that the incubation period of the auditory brainstem response (ABR) in women is shorter than that in men, but the incubation period and amplitude of postmenopausal women are the same as those of men of the same age (8). In addition, HL in women continues to decline after menopause, with hearing decline being more rapid after menopause than before (9). The difference of hearing level between males and females and the change of hearing level in perimenopausal women suggests a protective effect of estrogen on hearing. TS is a chromosome aberration caused by the deletion of all or part of the X chromosome. The main feature is ovarian hypoplasia, little or no endogenous estrogen production, often accompanied by ear and hearing problems. “Turner mice”, as a model of TS ear problems, showed otitis media and early HL, and the ABR of Turner mice showed progressive HL in high-frequency ranges, and was related to age (32, 33). Another study found that the hearing of female mice before menopause is better than that of male mice, but this advantage is weakened after menopause, which indicates that estrogen has certain effects on hearing (7, 37). However, in the current study there was no correlation between hearing loss and estrogen levels in TS patients. The reason for this is unclear but may relate to all patients receiving HRT. In addition, TS patients often display additional different non-sex chromosomal abnormalities that may affect hearing associated genes, although this association required further investigation.

TS patients often also present with endocrine abnormalities, metabolic diseases and abnormal bone metabolism. Approximately 30–50% of TS patients suffer from thyroiditis or hypothyroidism, 40–45% are positive for peroxidase antibodies, and have an increased risk for development of autoimmune diseases (38–40). Congenital and acquired hypothyroidism is a common cause of SNHL, mainly manifested by changes in audiological examinations such as brainstem auditory evoked potentials and hearing thresholds, these changes will occur after timely thyroid hormone replacement therapy (12). Animal models also found that reduced thyroid hormone secretion affects the development of the inner ear, leading to changes in the structure of the inner ear cochlea, which leads to SNHL (41–43). At present, there is no relevant research on the relationship between hearing level and thyroid function in patients with TS, and our data demonstrate no association between HL and thyroid function, further studies are needed to assess the relationship.

Metabolic syndrome is more common in TS, and it has been reported that about 50% of adolescents with TS have hypercholesterolemia (4, 44). Previous studies have shown that lipid profiles and obesity are risk factors for HL (13, 14, 45). Metabolic syndrome and its related factors are risk factors for HL. With an increase in the number of related factors, such as abdominal obesity, hypertension, increased fasting blood glucose, in-creased TG level and decreased HDL-C level, the risk of HL will increase, and the number of metabolic syndrome related factors is significantly correlated with hearing threshold (46–48). It has also been shown that patients with mosaic karyotypes are more likely to develop dyslipidemia than patients with other karyotypes, while patients with circular chromosomes are more likely to develop metabolic disorders (22). Alvarez-Nava et al. (49) found that metabolic syndrome was a risk factor for SNHL in adult patients with TS and that reducing the severity of metabolic syndrome may help reduce the progression of SNHL. Our study compared the blood lipid, insulin levels, and BMI of the TS between the normal hearing group and the hearing loss group, and found that TS with HL was not associated with insulin resistance, obesity, and dyslipidemia, may be related to lifestyle or drug treatment interventions, such as metformin, metabolic abnormalities, and further studies are needed to explain the potential pathological mechanism between metabolic abnormalities and SNHL.

The occurrence time of abnormal bone metabolism of TS is about 20–30 years earlier than that of normal women, and the risk of fracture is about 25% higher than that of normal people (50, 51). Previous studies have found that osteoporosis and low BMD can lead to HL, which may be associated with abnormal bone metabolism in the temporal bone, including loss of bone mass in the ossicular chain in the middle ear and osseous structures in the inner ear such as the bone labyrinth (15–17, 52). Yueniwati (53) found that BMD of cochlea, ossicles, femur or spine was negatively correlated with hearing threshold, indicating that abnormal bone metabolism may lead to HL. Current studies have found that decreased HL and BMD increase the risk of fracture in women with TS (54, 55), while no study has confirmed that decreased BMD in women with TS increases the risk of HL. Previous studies have found that HRT can effectively improve the BMD levels of the whole body, femoral neck and hip in TS patients (56). In this study, it was found that after HRT in TS patients, there was no statistical difference in BMD between the normal hearing group and the hearing loss group, suggesting that HRT can improve BMD of patients to a certain extent, but there was no significant improvement in hearing level of patients. Therefore, hearing abnormalities in TS patients cannot be explained only by insufficient estrogen level, and it may be necessary to expand the sample size and conduct grouping studies to explore the correlation between clinical phenomena of HL and related etiology in TS.

## 5. Conclusion

TS is often manifested as age-related MFHL and HFHL, and the incidence of HL increases with age. Compared with other karyotypes, patients with 45, X haplotype have an increased risk of MFHL; karyotype or *SHOX* gene can be used as a predictor of hearing problems in TS. For patients with confirmed TS, endocrinologists should pay attention to HL, conduct regular (yearly) endoscopic examination and hearing monitoring, strengthen patient education, and reduce the impact of TS on their study, psychology and quality of life.

## Data availability statement

The original contributions presented in the study are included in the article/supplementary material, further inquiries can be directed to the corresponding author.

## Ethics statement

The study was conducted in accordance with the Declaration of Helsinki and approved by the Institutional Review Board (or Ethics Committee) of Guangzhou Women and Children's Medical Center (protocol code 2016042019 and May 2016). Informed consent was obtained from all subjects involved in the study. Written informed consent to participate in this study was provided by the participants' legal guardian/next of kin.

## Author contributions

Conceptualization: LL. Methodology: LL and XW. Formal analysis: HL, XW, SQ, FL, and YC. Data curation and writing—original draft preparation: HL. Writing—review and editing and supervision: LL and GEL. Funding acquisition: GEL. All authors have read and agreed to the published version of the manuscript.

## References

[1] Davenport ML. Approach to the Patient with Turner Syndrome. J Clin Endocrinol Metab. (2010) 95:1487–95. 10.1210/jc.2009-0926 20375216

[2] Cameron-Pimblett A La Rosa C King T Davies MC Conway GS. The Turner syndrome life course project: Karyotype-phenotype analyses across the lifespan. Clin Endocrinol (Oxf). (2017) 87:532–38. 10.1111/cen.13394 28617979

[3] Gravholt CH Andersen NH Conway GS Dekkers OM Geffner ME Klein KO. Clinical practice guidelines for the care of girls and women with Turner syndrome: proceedings from the 2016 Cincinnati International Turner Syndrome Meeting. Eur J Endocrinol. (2017) 177:G1–70. 10.1530/EJE-17-0430 28705803

[4] Gravholt CH Viuff MH Brun S Stochholm K Andersen NH. Turner syndrome: mechanisms and management. Nat Rev Endocrinol. (2019) 15:601–14. 10.1038/s41574-019-0224-4 31213699

[5] Bonnard A Hederstierna C Bark R Hultcrantz M. Audiometric features in young adults with Turner syndrome. Int J Audiol. (2017) 56:650–56. 10.1080/14992027.2017.1314559 28420278

[6] Hederstierna C Hultcrantz M Rosenhall U. Estrogen and hearing from a clinical point of view; characteristics of auditory function in women with Turner syndrome. Hear Res. (2009) 252:3–08. 10.1016/j.heares.2008.11.006 19095053

[7] Bonnard A Bark R Hederstierna C. Clinical update on sensorineural hearing loss in Turner syndrome and the X-chromosome. Am J Med Genet C Semin Med Genet. (2019) 181:18–24. 10.1002/ajmg.c.31673 30632288

[8] McFadden D Hsieh MD Garcia-Sierra A Champlin CA. Differences by sex, ear, and sexual orientation in the time intervals between successive peaks in auditory evoked potentials. Hear Res. (2010) 270:56–64. 10.1016/j.heares.2010.09.008 20875848 PMC2997906

[9] Svedbrant J Bark R Hultcrantz M Hederstierna C. Hearing decline in menopausal women–a 10-year follow-up. Acta Otolaryngol. (2015) 135:807–13. 10.3109/00016489.2015.1023354 25891312

[10] King KA Makishima T Zalewski CK Bakalov VK Griffith AJ Bondy CA. Analysis of auditory phenotype and karyotype in 200 females with Turner syndrome. Ear Hear. (2007) 28:831–41. 10.1097/AUD.0b013e318157677f 17982369

[11] Lim D Hassani S Lupton K Gault EJ Wynne D Clement W. Prevalence, risk factors and management strategies for otological problems in girls with Turner syndrome. Acta Paediatr. (2020) 109:2075–83. 10.1111/apa.15128 31811789

[12] Anjana Y Vaney N Tandon OP Madhu SV. Functional status of auditory pathways in hypothyroidism: evoked potential study. Indian J Physiol Pharmacol. (2006) 50:341–49. 17402263

[13] Lee JS Kim DH Lee HJ Kim HJ Koo JW Choi HG et al. Lipid profiles and obesity as potential risk factors of sudden sensorineural hearing loss. PLoS ONE. (2015) 10:e122496. 10.1371/journal.pone.0122496 25860024 PMC4393091

[14] Mohammed AA. Lipid profile among patients with sudden sensorineural hearing loss. Indian J Otolaryngol Head Neck Surg. (2014) 66:425–28. 10.1007/s12070-014-0744-0 26396956 PMC4571478

[15] Curhan SG Stankovic K Halpin C Wang M Eavey RD Paik JM. Osteoporosis, bisphosphonate use, and risk of moderate or worse hearing loss in women. J Am Geriatr Soc. (2021) 69:3103–13. 10.1111/jgs.17275 34028002 PMC8595486

[16] Yoo JI Park KS Seo SH Park HW. Osteoporosis and hearing loss: findings from the Korea National Health and Nutrition Examination Survey 2009-2011. Braz J Otorhinolaryngol. (2020) 86:332–38. 10.1016/j.bjorl.2018.12.009 30827872 PMC9422524

[17] Juneja MK Munjal S Sharma A Gupta AK Bhadada S. Audiovestibular functioning of post-menopausal females with osteoporosis and osteopenia. J Otol. (2021) 16:27–33. 10.1016/j.joto.2020.07.007 33505447 PMC7814074

[18] Chadha S Kamenov K Cieza A. The world report on hearing, 2021. Bull World Health Organ. (2021) 99:242. 10.2471/BLT.21.285643 33953438 PMC8085630

[19] Favier V Vincent C Bizaguet E Bouccara D Dauman R Frachet B. French Society of ENT (SFORL) guidelines (short version): Audiometry in adults and children. Eur Ann Otorhinolaryngol Head Neck Dis. (2018) 135:341–47. 10.1016/j.anorl.2018.05.009 29929777

[20] Kubba H Smyth A Wong SC Mason A. Ear health and hearing surveillance in girls and women with Turner's syndrome: recommendations from the Turner's Syndrome Support Society. Clin Otolaryngol. (2017) 42:503–07. 10.1111/coa.12750 27614170

[21] Hederstierna C Hultcrantz M Rosenhall U. A longitudinal study of hearing decline in women with Turner syndrome. Acta Otolaryngol. (2009) 129:1434–41. 10.3109/00016480902741962 19922094

[22] Fiot E Zenaty D Boizeau P Haignere J Dos SS Leger J. X chromosome gene dosage as a determinant of congenital malformations and of age-related comorbidity risk in patients with Turner syndrome, from childhood to early adulthood. Eur J Endocrinol. (2019) 180:397–406. 10.1530/EJE-18-0878 30991358

[23] Bergamaschi R Bergonzoni C Mazzanti L Scarano E Mencarelli F Messina F et al. Hearing loss in Turner syndrome: results of a multicentric study. J Endocrinol Invest. (2008) 31:779–83. 10.1007/BF03349257 18997489

[24] Miguel-Neto J Carvalho AB Marques-de-Faria AP Guerra-Junior G Maciel-Guerra AT. New approach to phenotypic variability and karyotype-phenotype correlation in Turner syndrome. J Pediatr Endocrinol Metab. (2016) 29:475–79. 10.1515/jpem-2015-0346 26812779

[25] Dumancic J Kaic Z Varga ML Lauc T Dumic M Milosevic SA. Characteristics of the craniofacial complex in Turner syndrome. Arch Oral Biol. (2010) 55:81–8. 10.1016/j.archoralbio.2009.10.008 19942211

[26] Chan KC Wang PC Wu CM Ho WL Lo FS. Otologic and audiologic features of ethnic Chinese patients with Turner syndrome in Taiwan. J Formos Med Assoc. (2012) 111:94–100. 10.1016/j.jfma.2010.11.001 22370288

[27] Verver EJ Freriks K Thomeer HG Huygen PL Pennings RJ Alfen-van DVA. Ear and hearing problems in relation to karyotype in children with Turner syndrome. Hear Res. (2011) 275:81–8. 10.1016/j.heares.2010.12.007 21147207

[28] Bazilio M Santos A Almeida FG Frota S Guimaraes M Ribeiro MG. Association between cytogenetic alteration and the audiometric profile of individuals with Turner syndrome. Braz J Otorhinolaryngol. (2020) 87:728–32. 10.1016/j.bjorl.2020.03.005 32402566 PMC9422574

[29] Morimoto N Tanaka T Taiji H Horikawa R Naiki Y Morimoto Y. Hearing loss in Turner syndrome. J Pediatr. (2006) 149:697–701. 10.1016/j.jpeds.2006.06.071 17095347

[30] Barrenas ML Nylen O Hanson C. The influence of karyotype on the auricle, otitis media and hearing in Turner syndrome. Hear Res. (1999) 138:163–70. 10.1016/S0378-5955(99)00162-8 10575123

[31] Hultcrantz M Sylven L Borg E. Ear and hearing problems in 44 middle-aged women with Turner's syndrome. Hear Res. (1994) 76:127–32. 10.1016/0378-5955(94)90094-9 7928705

[32] Hultcrantz M Stenberg AE Fransson A Canlon B. Characterization of hearing in an X,0 'Turner mouse'. Hear Res. (2000) 143:182–88. 10.1016/S0378-5955(00)00042-3 10771195

[33] Stenberg AE Wang H Sahlin L Stierna P Enmark E Hultcrantz M. Estrogen receptors alpha and beta in the inner ear of the 'Turner mouse' and an estrogen receptor beta knockout mouse. Hear Res. (2002) 166:1–08. 10.1016/S0378-5955(02)00310-6 12062753

[34] Ros C Tercero A Alobid I Balasch J Santamaria J Mullol J. Hearing loss in adult women with Turner's syndrome and other congenital hypogonadisms. Gynecol Endocrinol. (2014) 30:111–16. 10.3109/09513590.2013.856002 24256370

[35] Barrenas M Landin-Wilhelmsen K Hanson C. Ear and hearing in relation to genotype and growth in Turner syndrome. Hear Res. (2000) 144:21–8. 10.1016/S0378-5955(00)00040-X 10831862

[36] Bois E Nassar M Zenaty D Leger J Van Den Abbeele T Teissier N. Otologic disorders in Turner syndrome. Eur Ann Otorhinolaryngol Head Neck Dis. (2018) 135:21–4. 10.1016/j.anorl.2017.08.006 28941966

[37] Guimaraes P Zhu X Cannon T Kim S Frisina RD. Sex differences in distortion product otoacoustic emissions as a function of age in CBA mice. Hear Res. (2004) 192:83–9. 10.1016/j.heares.2004.01.013 15157966

[38] Mortensen KH Cleemann L Hjerrild BE Nexo E Locht H Jeppesen EM. Increased prevalence of autoimmunity in Turner syndrome–influence of age. Clin Exp Immunol. (2009) 156:205–10. 10.1111/j.1365-2249.2009.03895.x 19298606 PMC2759466

[39] Mohamed S Elkhidir I Abuzied A Noureddin A Ibrahim G Mahmoud A. Prevalence of autoimmune thyroid diseases among the Turner Syndrome patients: meta-analysis of cross sectional studies. Bmc Res Notes. (2018) 11:842. 10.1186/s13104-018-3950-0 30486859 PMC6264051

[40] Elsheikh M Wass JA Conway GS. Autoimmune thyroid syndrome in women with Turner's syndrome–the association with karyotype. Clin Endocrinol. (2001) 55:223–26. 10.1046/j.1365-2265.2001.01296.x 11531929

[41] Knipper M Richardson G Mack A Muller M Goodyear R Limberger A. Thyroid hormone-deficient period prior to the onset of hearing is associated with reduced levels of beta-tectorin protein in the tectorial membrane: implication for hearing loss. J Biol Chem. (2001) 276:39046–52. 10.1074/jbc.M103385200 11489885

[42] Karolyi IJ Dootz GA Halsey K Beyer L Probst FJ Johnson KR. Dietary thyroid hormone replacement ameliorates hearing deficits in hypothyroid mice. Mamm Genome. (2007) 18:596–608. 10.1007/s00335-007-9038-0 17899304

[43] Ng L Hernandez A He W Ren T Srinivas M Ma M. A protective role for type 3 deiodinase, a thyroid hormone-inactivating enzyme, in cochlear development and auditory function. Endocrinology. (2009) 150:1952–60. 10.1210/en.2008-1419 19095741 PMC2659284

[44] Ross JL Feuillan P Long LM Kowal K Kushner H Cutler GJ. Lipid abnormalities in Turner syndrome. J Pediatr. (1995) 126:242–45. 10.1016/S0022-3476(95)70551-1 7844670

[45] Chen C Wang M Fan Z Zhang D Lyu Y Wang H. Correlations between the pathogenesis and prognosis of sudden sensorineural hearing loss and blood lipid. Zhonghua Er Bi Yan Hou Tou Jing Wai Ke Za Zhi. (2015) 50:793–98. 26696470

[46] Rim HS Kim MG Park DC Kim SS Kang DW Kim SH. Association of metabolic syndrome with sensorineural hearing loss. J Clin Med. (2021) 10:4866. 10.3390/jcm10214866 34768385 PMC8584388

[47] Sun YS Fang WH Kao TW Yang HF Peng TC Wu LW. Components of metabolic syndrome as risk factors for hearing threshold shifts. PLoS ONE. (2015) 10:e134388. 10.1371/journal.pone.0134388 26247614 PMC4527724

[48] Shim HS Shin HJ Kim MG Kim JS Jung SY Kim SH. Metabolic syndrome is associated with hearing disturbance. Acta Otolaryngol. (2019) 139:42–7. 10.1080/00016489.2018.1539515 30664389

[49] Alvarez-Nava F Racines-Orbe M Witt J Guarderas J Vicuna Y Estevez M. Metabolic syndrome as a risk factor for sensorineural hearing loss in adult patients with Turner Syndrome. Appl Clin Genet. (2020) 13:25–35. 10.2147/TACG.S229828 32021381 PMC6971290

[50] Nadeem M Roche EF. Bone health in children and adolescent with Turner syndrome. J Pediatr Endocrinol Metab. (2012) 25:823–33. 10.1515/jpem-2012-0088 23426807

[51] Bakalov VK Bondy CA. Fracture risk and bone mineral density in Turner syndrome. Rev Endocr Metab Disord. (2008) 9:145–51. 10.1007/s11154-008-9076-2 18415020

[52] Kim SY Kong IG Lim H Choi HG. Increased risk of sudden sensory neural hearing loss in osteoporosis: a longitudinal follow-up study. J Clin Endocrinol Metab. (2018) 103:3103–09. 10.1210/jc.2018-00717 29846624

[53] Yueniwati Y. The significant correlation between the density of the cochlea otic capsule and spine in hearing loss patients. Indian J Otolaryngol Head Neck Surg. (2019) 71:1163–68. 10.1007/s12070-018-01580-z 31750143 PMC6841858

[54] Satar B Ozkaptan Y Surucu HS Ozturk H. Ultrastructural effects of hypercholesterolemia on the cochlea. Otol Neurotol. (2001) 22:786–89. 10.1097/00129492-200111000-00012 11698796

[55] Augoulea A Zachou G Lambrinoudaki I. Turner syndrome and osteoporosis. Maturitas. (2019) 130:41–9. 10.1016/j.maturitas.2019.09.010 31706435

[56] Li L Qiu X Lash GE Yuan L Liang Z Liu L. Effect of hormone replacement therapy on bone mineral density and body composition in Chinese adolescent and young adult Turner syndrome patients. Front Endocrinol. (2019) 10:377. 10.3389/fendo.2019.00377 31244781 PMC6582219

